# Predictors and Clinical Outcomes in Empyema Thoracis Patients Presenting to the Emergency Department Undergoing Video-Assisted Thoracoscopic Surgery

**DOI:** 10.3390/jcm8101612

**Published:** 2019-10-03

**Authors:** Yuan-Ming Tsai, Nikita Gamper, Tsai-Wang Huang, Shih-Chun Lee, Hung Chang

**Affiliations:** 1 Division of Thoracic Surgery, Department of Surgery, Tri-Service General Hospital, National Defense Medical Center, No. 325, Section 2, Cheng-Kung Road, Taipei 114, Taiwan; umymt@leeds.ac.uk (Y.-M.T.);; 2 School of Biomedical Sciences, Faculty of Biological Sciences, University of Leeds, Leeds LS2 9JT, UK; N.Gamper@leeds.ac.uk

**Keywords:** pneumonia, pleural empyema, chest pain, video-assisted thoracoscopic surgery, emergency department, mortality

## Abstract

Background: Video-assisted thoracoscopic surgery (VATS) is widely used for the treatment of empyema. We evaluated clinical symptoms, laboratory examinations, and thoracentesis to assess patients in the emergency department (ED) with empyema thoracis, undergoing VATS to identify predictors of adverse outcomes. Methods: This retrospective study was conducted by reviewing records of ED patients with pleural empyema admitted for VATS from January 2007 to June 2014. Demographic data, clinical symptoms, and laboratory examinations were compared for survivors (Group I) and non-survivors (Group II). Logistic regression analysis was used to identify parameters related to postoperative mortality. Results: From 380 patients, 7.6% (*n* = 29) died postoperatively. Survivors and non-survivors exhibited differences in age, gender, presence of cough, dyspnea, chest pain, empyema stage, cerebrovascular disease, malignancy, the glucose level of pleural fluid, serum hemoglobin, platelet count, blood urea nitrogen, and potassium levels. The logistic analysis demonstrated that the most significant factor related to the postoperative morbidity is chest pain (*p* = 0.018). Conclusions: VATS could be a safe option for pediatric and geriatric patients. Age does not appear to affect postoperative mortality. A high degree of awareness is essential for perioperative management and early surgical treatment when ED patients present with the clinical symptom of chest pain.

## 1. Introduction

Empyema thoracis is a collection of purulent fluid in the pleural space that mainly occurs with pneumonia or secondary to chest trauma/surgery. Approximately half of the patients with pneumonia develop pleural effusion, and 5–10% of patients may develop empyema thoracis after antibiotic treatment [1]. It evolves from stage I (exudative), through stage II (fibrinopurulent), to stage III (organized) for 3 to 6 weeks. Patients may have a fever, chest pain, and cough in the early stage, whereas dyspnea may occur in the late stage. Treatment includes antibiotics, chest tube drainage, surgical intervention with video-assisted thoracoscopic surgery (VATS), or open thoracotomy. However, empyema thoracis is still a common problem, with high morbidity and mortality rates as a result of suboptimal management [2]. Despite the use of antibiotics and different pneumococcal vaccines in clinical care, and improvement of the minimally invasive surgical techniques, empyema remains the most common complication of pneumonia with a reported mortality rate between 10% and 20% [3]. Recent consensus guidelines show that the use of a chest tube as the first-line treatment still needs further examination [4,5]. A cohort study in 2018 characterized the higher readmission and reintervention rates in patients with thoracic empyema treated with chest tubes, and suggested earlier surgical intervention [5]. Adequate treatment of thoracic empyema is stage-dependent, and surgical intervention will be considered when antibiotic therapy and fluid drainage do not achieve infection control successfully, along with good re-expansion of the lung [6]. Since the progression of thoracic empyema is fluent, stratification by stage and treatment depend on the surgeon’s experience and judgment. Although a systematic review demonstrated that treatment with VATS had superior outcomes for pleural empyema, even as the first-line management [5,7], no consensus currently exists about the timing for choosing VATS for the initial treatment. This study focused on patients presenting to the emergency department (ED) with suspected empyema thoracis. Knowing the risks for death related to treatment with VATS may enable surgeons to optimize therapeutic strategies for definitive surgical management to avoid postoperative complications, and even to reduce mortality.

## 2. Material and Methods

During the study period (January 2007 to June 2014), a total of 380 patients presenting to our ED with empyema thoracis were admitted for further evaluation and treatment. These patients received immediate, detailed clinical and serum examinations. Before diagnostic thoracentesis, the disease status was assessed via chest X-ray or computed tomography (CT). Patients were prescribed empiric broad-spectrum intravenous antibiotics for the suspicion of thoracic empyema. Surgical intervention with VATS was indicated in the presence of persistent fever, residual fluid after drainage, sepsis, and worsening of the patient’s status with septations or loculations. All patients were operated on under general anesthesia with the two- or three-portal procedure, based on the CT scan findings. The process is described as follows. The first port was created at the site with pronounced collection. Once the pleural cavity was entered, contents were suctioned, and other ports were created under visual observation to avoid injury to the lung parenchyma. Instruments could be used between the camera and working ports, and no rib retraction was applied in these patients. A specimen was collected for microbiological tests, and inflammatory peel was stripped using both sharp and blunt dissection. After completion of debridement, chest wall bleeding was resolved with electrocautery, and the status of the affected lung and its expansion was assessed. At the end of the procedure, one or two chest tubes were inserted under thoracoscopic visualization. Patients were discharged after removal of the chest tube, with pleural drainage of less than 200 mL per day and completion of intravenous antibiotics.

Patients who initially presented to the ED with septic shock, ventilator dependence and cardiopulmonary distress, those lost to follow-up, and children with immunodeficiency and cerebral palsy associated with swallowing disorders were excluded from this study. Images were judged by a radiologist during routine radiological assessment. Two thoracic surgeons reviewed these findings and the corresponding images before surgical treatment. The images were classified as follows: stage I referred to fluid accumulation without loculations; stage II had fibrinous septations with loculations and the presence of pus; stage III had multiple loculations and entrapped the underlying lung [8]. For the study, patients were divided into two groups: survivors (Group I) or non-survivors (Group II). The following data were collected for the analysis of risk factors of postoperative mortality: Age, gender, current smoking, comorbidities (chronic obstructive pulmonary disease, hypertension, diabetes mellitus, renal insufficiency, cerebrovascular disease and malignancy), clinical presentation (fever, cough, dyspnea and chest pain), biological samples (blood and pleural effusion), duration of surgery (time from skin incision to skin closure), and location and stage of empyema thoracis. The hospital’s ethics committee granted the ethical approval for this study, and informed consent was waived due to the retrospective study design.

### Statistical Analysis

Categorical variables were compared by using χ^2^ or Fisher’s exact analyses. Continuous variables were compared by using Mann–Whitney U analysis. A multivariate logistic regression analysis was performed to assess the association between the variables, with statistically significant differences between the two groups. A two-sided *p* value < 0.05 was used as the criterion for statistical significance. Statistical analyses were performed using SPSS software ver. 22.0 (IBM, Armonk, NY, USA).

## 3. Results

A total of 380 patients were included in the analysis: 287 (75.5%) males and 93 (24.5%) females. Sixty-two percent were 18 to less than 65 years old, 33% were aged 65 and above, and 5% were less than 18 years old. The mean age was 55.4 ± 20.0 years. Twenty-nine (7.6%) patients died during the 30 days following the operation. The demographic and clinical characteristics of the two groups are displayed in Table 1. One hundred and ninety-one (50.3%) patients were current smokers. The most prevalent comorbidity was hypertension (33.9%), followed by diabetes (21.3%), chronic obstructive pulmonary disease (17.1%), cerebrovascular disease (10.3%), malignancy (7.9%), and chronic renal insufficiency (5.8%). The common symptoms at ED were dyspnoea (45.3%), fever (45.0%), chest pain (35.3%), and cough (35.0%). Two hundred and thirty (60.5%) patients had right-sided thoracic empyema, and 34 (8.9%) patients had stage III empyema. The average operating time was 125.1 ± 91.8 min. There were significant differences in age (patients ≥65 y of age), gender, cerebrovascular disease, malignancy, dyspnea, chest pain, cough, and stage of empyema thoracis between the two groups.

Table 2 shows data from laboratory examinations. A pathogen was identified in 90 out of 380 (23.7%) patients with thoracic empyema in the ED and 9 (31.0%) patients had a positive culture in Group II: methicillin-resistant *Staphylococcus aureus* in five (55.6%), *Staphylococcus aureus* in three (33.3%), and *Klebsiella pneumonia* in one (11.1%). However, there was no statistically significant difference between the groups.

Table 3 shows the results of the diagnostic thoracentesis. The non-survivor group had a significantly higher pleural glucose level (144 ± 121 vs. 79 ± 127 mg/dL; *p* = 0.041). No significant difference between the groups was found in pH value, white cell count (WBC), and lactate dehydrogenase (LDH) levels in the pleural fluid. Laboratory examination of the blood revealed that the hemoglobin level and platelet count were significantly lower in non-survivors (hemoglobin: 9.60 ± 1.86 vs. 11.53 ± 2.23 g/dL, *p* = 0.001; platelets: 202.00 ± 131.91 vs. 294.11 ± 15.77 10^9^/L, *p* = 0.017). Blood urea nitrogen (BUN) and potassium levels were significantly higher in non-survivors (BUN: 32.50 ± 25.47 vs. 20.88 ± 21.77 mg/dL, *p* = 0.049; potassium: 4.10 ± 0.78 vs. 3.79 ± 0.55, *p* = 0.046). There was no significant difference between groups in serum WBC, creatinine, and sodium levels. Multiple logistic regression analysis was performed using the factors described in the previous sections. The results implied that cerebrovascular disease (*p* = 0.001), chest pain (*p* = 0.018), and serum potassium level (*p* = 0.023) were independent risk factors for mortality, as shown in Table 4.

## 4. Discussion

The incidence of pleural empyema increased at a rate of 2.8% per year from 1987 to 2004, and a twofold increase occurred in the United States [9,10]. Review articles also showed the incidence of empyema ranging from 0.9 to 12.5 per 100,000 population among children aged <19 years in Utah (USA) (1994–2007) and Australia (1998–2010), and was higher in those aged >65 years (3.5–4.8 per 100,000) than other adult age groups in 1996–2001 and 2006–2009 [11]. Likewise, the significant increase in the incidence of adult empyema, mainly in young adults (aged 18–50 years), was from 7.6% in 1996–2001 to 14.9% in 2005–2011, and a 23% increase has been observed in Canada (ages 40–54 years) [12]. However, there was no improvement in mortality over the study period [3]. Empyema thoracis remains challenging for thoracic surgeons, with a high mortality rate between 5.4% to 18.3% [5]. Cases are also seen in children with delayed referral to ED with persistent fever, cough, and several courses of broad-spectrum antibiotics by community physicians. In this 7-year single-center cohort study, we analyze the outcomes of patients presenting to the ED with symptoms and signs of empyema thoracis. Although they had VATS treatment during hospitalization, 7.6% of those patients died within 30 days after surgery, in or out of the hospital. The mortality may be associated with inadequate source control, underlying comorbidities, or delays in providing definitive therapy. Therefore, identification of risk factors and early surgical intervention in pleural space infections may help improve overall survival rates and reduce the burden on hospitals.

Age was the leading risk factor for mortality, with a rate of 16.1% among elderly patients (≥65 years) [10]. Immunity is different in older and younger persons, such that elderly individuals do not respond to immune challenge as robustly as the young [13]. Older people may present with atypical symptoms, particularly decreased severity of chest pain and fever than younger patients in the ED, which makes the early diagnosis of thoracic empyema difficult [14]. Additionally, higher age and a higher rate of comorbidities are related to functional decline, which possibly results in older patients with thoracic empyema having higher mortality rates [15]. However, recent findings have shown that multi-morbidity, rather than age, is the primary risk factor for a fatal outcome in all patients undergoing surgical treatment for pleural empyema [3]. Besides, initial chest tube management has been reported to have a higher failure rate, with the necessity of reintervention during hospitalization [5,16,17]. There is no doubt that the advent of VATS has made it possible to treat early-stage empyema with less morbidity. However, no evidence exists regarding the surgical approach (thoracotomy vs. VATS) for stage III empyema [6,18]. Although management of chronic stage III empyema remains a challenge for the thoracic surgeon, VATS has effectiveness in treating multiloculated and chronic empyema. Our study indicated that comorbidity with cerebrovascular disease is related to mortality, whereas age or empyema stage was not a risk factor for mortality. This may be the result of correct and targeted antibiotic treatment, and improvement of minimally invasive surgical techniques in recent decades.

Clinical symptoms have been suggested as one of the means to estimate the stage of empyema, as patients in empyema stage II and III may experience shortness of breath which is caused by pleural adhesion and decreased expansion of the lung [7,19]. In our study, there was no statistical significance in the symptoms of fever, cough, and dyspnea, but chest pain was the significant risk factor differentiating the two groups. This surprising risk factor may be induced by compression of the intercostal nerves [20].

Evaluating and diagnosing through early identification of the microbial etiologies would guide early targeted antimicrobial therapy and improve clinical outcomes [21]. Our data showed the *S. aureus* and methicillin-resistant *S. aureus* were the most frequently identified pathogen in the non-survivor group, which is in agreement with published data on the etiology of pleural empyema undergoing surgical treatment [22,23,24]. It is important to note that *Viridans streptococci* and *Mycobacteria tuberculosis* are the most common cause of empyema in the survivor group, which may be easily overlooked. *Viridans streptococci* are most prevalent in the oral cavity and upper respiratory tract, and are the main etiologic agents of empyema with a less frequent productive cough and more frequent bed-ridden status [25,26]. Although uncommon, *Viridans streptococci* are emerging as a cause of community-acquired respiratory tract infection, with an incidence of 3% to 15% in a few studies [26]. Tuberculous empyema has been reported in nearly 40% of thoracic empyema in India [27], and there is an increasing incidence of *Mycobacterium tuberculosis* in the United Kingdom and elsewhere in Europe, so it is imperative that all patients with empyema are routinely screened [21,28].

Through the laboratory analysis of blood and pleural fluid, it is possible to provide much useful information for the cause of pleural effusions. A long observation period study in the United Kingdom showed that multiple preoperative laboratory results were associated with early postoperative mortality, without data regarding serum potassium [21]. Although the pleural glucose concentration, serum hemoglobin, platelets, BUN, and potassium levels were significantly different in our study, further logistic analysis showed only the serum potassium level was an independent risk factor for postoperative mortality. Dyskalemia is a condition which occurs in patients with severe sepsis as a result of inadequate drainage and inefficient control of infection.

The indication for and timing of surgical intervention in the management of empyema remains controversial, especially in children. An important consideration to perform any surgical procedure is to weigh the benefits against the risks in the appropriate patients, to improve overall outcomes. In this study, we evaluated the possible risk factors of empyema thoracis treatment by VATS from the ED patients’ records. Early surgical intervention and multidisciplinary care can decrease the mortality rate, especially for those who present to ED with the symptom of chest pain.

The strengths of this study are the long observational period and the single-center design, with a large number of patients. However, the study also has several limitations. First, our center is a large university teaching hospital, and many patients were already pretreated and secondarily referred by primary physicians or other hospitals, and many details of the initial treatment are now impossible to retrieve. Second, there is no standardized and uniform management algorithm for the timing of surgical intervention when initial treatment fails. Additionally, the treatment by tube thoracostomy for preoperative management in our cohort of patients was not analyzed.

## 5. Conclusions

In conclusion, this study demonstrates several important features of ED patients presenting with empyema. VATS is a safe and effective procedure in all ages, and in stage III thoracic empyema patients. There is a trend of *Viridans streptococci* and *Mycobacterium tuberculosis* as emerging pathogens in the pathogenesis of empyema. Most importantly, the clinical symptom of chest pain is a reliable factor to predict an adverse outcome of medical therapy when the patient is first admitted via ED. In particular, the post-operative outcome may be related to cerebrovascular disease and the serum potassium level. However, further large-scale studies are warranted to support our observations.

## Figures and Tables

**Table 1:** Demographic data of patients.

Variables	Group I: Survivors (*n* = 351)	Group II: Non-Survivors (*n* = 29)	*p* Value *
Age (years)	54.3 ± 20.1	68.8 ± 12.6	<0.001
<18	20 (100.0%)	0 (0.0%)	0.114
≥18 and <65	221 (94.44%)	13 (5.56%)	0.638
≥65	110 (87.30%)	16 (12.70%)	0.039
Gender			0.006
Male	272 (94.77%)	15 (5.23%)	
Female	79 (84.95%)	14 (15.05%)	
Current smoking	177 (92.6%)	14 (7.4%)	0.849
Comorbidity			0.178
Hypertension	119 (92.2%)	10 (7.8%)	1.000
Diabetes mellitus	71 (87.7%)	10 (12.3%)	0.096
Chronic obstructive pulmonary disease	58 (89.2%)	7 (10.8%)	0.306
Cerebrovascular disease	32 (82.1%)	7 (17.9%)	0.020
Renal insufficiency	20 (90.9%)	2 (9.1%)	0.680
Malignancy	23 (76.7%)	7(23.3%)	0.004
Symptoms			0.552
Fever	163 (95.3%)	8 (4.7%)	0.054
Dyspnea	153 (89.0%)	19 (11.0%)	0.031
Chest pain	129 (96.3%)	5 (3.7%)	0.042
Cough	129 (97.0%)	4 (3.0%)	0.014
Location			0.316
Right	210 (91.3%)	20 (8.7%)	
Left	141 (94.0%)	9 (6.0%)	
Stage			0.025
I-II	323(93.4%)	23(6.6%)	
III	28(82.4%)	6(17.6%)	
Operative time (mins)	125.4 ± 88.9	121.2 ± 123.3	0.813

* Significance level: *p* < 0.05.

**Table 2:** Organisms identified from the pleural fluid.

Organisms	Group I: Survivors (*n* = 351)	Group II: Non-Survivors (*n* = 29)	*p* Value *
**Positive**	81 (90.0%)	9 (10.0%)	0.326
*Staphylococcus aureus*	8 (72.7%)	3 (27.3%)	
Methicillin-resistant *Staphylococcus aureus*	5 (50.0%)	5 (50.0%)	
*Klebsiella pneumoniae*	18 (94.7%)	1 (5.3%)	
Extended-spectrum beta-lactamase-producing *Klebsiella pneumoniae*	1 (100.0%)	0 (0.0%)	
*Viridans streptococci*	24 (100.0%)	0 (0.0%)	
*Mycobacteria tuberculosis*	23 (100.0%)	0 (0.0%)	
*Pseudomonas aeruginosa*	2 (100.0%)	0 (0.0%)	

* Significance level: *p* < 0.05.

**Table 3:** Pleural fluid and laboratory findings analysis.

Variables	Group I: Survivors (*n* = 351)	Group II: Non-Survivors (*n* = 29)	*p* Value *
**Pleural effusion**			
pH value	7.68 ± 0.59	7.79 ± 0.47	0.429
White cell count	9661 ± 25,886	31,909 ± 78,096	0.448
Glucose, mg/dL	79 ± 127	144 ± 121	0.041
Lactate dehydrogenase (LDH), units/L	1918 ± 3478	2000 ± 5805	0.927
**Blood**			
White cell count	15,010 ± 10,391	13,261 ± 6,615	0.373
Hemoglobin, g/dL	11.53 ± 2.23	9.60 ± 1.86	0.001
Platelet counts, 10^9^/L	294.11 ± 150.70	202.00 ± 131.91	0.017
Blood Urea Nitrogen, mg/dL	20.88 ± 21.77	32.50 ± 25.47	0.049
Creatinine, mg/dL	1.20 ± 1.23	1.67 ± 1.52	0.145
Sodium levels	134.58 ± 3.91	134.25 ± 5.53	0.759
Potassium levels	3.79 ± 0.55	4.10 ± 0.78	0.046

* Significance level: *p* < 0.05.

**Table 4:** Logistic regression coefficients (beta) and odds ratio estimates from the multivariate logistic regression models for factors affecting mortality.

Factors	Coefficient		*p* Value *	OR	95% Confidence Interval for B	
	B	Std. Error			Lower Bound	Upper Bound
Cerebrovascular disease	−0.341	0.085	0.001	0.74	–0.459	–0.122
Malignancy	–0.205	0.082	0.051	0.82	–0.325	0.001
Chest Pain	0.240	0.056	0.018	1.23	0.024	0.249
Serum Potassium level	–0.237	0.047	0.023	0.79	–0.201	–0.015

* Significance level: *p* < 0.05.
